# Medical News

**Published:** 1852-10

**Authors:** 


### MEDICAL NEWS.

PROGRESS of EPIDEMICS.—The cholera still claims its victims, and is making rapid advance towards our coasts. In Warsaw from fifty to sixty die daily; and on the 6th and 7th instant, it is said, 240 persons died.——At Posen, the mortality is very great, and in Dantzic, where it has also shown itself, four-fifths of those attacked perish in consequence.——Lamentable accounts have been received of the mortality among the inhabitants of the Trans-Vaal territory, Cape of Good Hope, arising from a contagious disease peculiar to the country, which has this season assumed an unusual degree of virulence. Upwards of 300 of the settlers have been cut off in one locality. The disease, it is said, is very rapid in its operation, generally ending in death within a few hours of the attack.——The small-pox continues to prevail in Jamaica; the yellow fever has much abated at Rio de Janeiro, and only a few cases now remain.——The official *Gazette* of Savoy states, that a deep incision in the trunk of the vine, near the root, cures the mangra, the disease which has been so destructive to vineyards this year.——There is a very sad report that the disease, which has ruined the potato crop, has extended to the wheat; its extent at present is undecided.——The drought has been very great all through the United States, and the crops have suffered severely in consequence. Latterly, however, there has been, as in this country, a succession of heavy thunder-storms, but the heat remains as intense as before. The public health throughout the country has suffered. All through the west and south a species of malignant cholera has prevailed extensively, while along the banks of the western rivers the Asiatic cholera, in a less mitigated form, has swept off multitudes of the inhabitants.—*Lond. Med. Times, Aug.* 21, 1852.

OBITUARY.—MR. HERBERT MAYO.—This well known author and surgeon died, a few days since, in Germany. Mr. Mayo was formerly lecturer on anatomy and physiology at King's College, and surgeon to the Middlesex Hospital. Of late years, however, he forsook the legitimate path of his profession, and became a mesmerist and a hydropath, on both which subjects he wrote extensively. His papers in the late *Medical Gazette* on the subject of mesmerism, exceeded all bounds, and many of his friends were fearful, at the time, that his mind had become wrecked. He subsequently embraced hydropathy, and retired to practise this heresy into Germany. Whilst in sound mind Mr. Mayo was undoubtedly an able writer and original thinker, and his lectures on Anatomy and Physiology were remarkable for clearness and beauty of style. His practical abilities as surgeon were great, but certainly physiology was his forte. His work on Physiology, although now superseded by more modern productions, will always be remembered with pleasure by those who read it. Mr. Mayo was somewhat conceited, but withal an amiable man. He was deficient, however, to a remarkable extent, in worldly wisdom; as an instance of which it may be mentioned, that whilst lecturing on Physiology, at King's College, he actually became a candidate for the professorship of the same science at University College, vacant by the resignation of Dr. Jones Quain; and then was astonished to find that the council of King's College did not approve of his conduct.—*London Lancet, Aug.* 28, 1852.

